# Overview and Regional Context of Alzheimer’s Disease in the Middle East and North Africa Region

**DOI:** 10.7759/cureus.83164

**Published:** 2025-04-29

**Authors:** Akum Dhillon, Jawad Fazal

**Affiliations:** 1 Department of Research and Health Innovation, Burjeel Medical City, Abu Dhabi, ARE; 2 Department of Neurology, Burjeel Medical City, Abu Dhabi, ARE

**Keywords:** alzheimer’s disease, burden, dementia, epidemiology, middle east, risk factors

## Abstract

Alzheimer's disease (AD) poses a significant global health challenge, particularly in the Middle East and North Africa (MENA) due to unique socio-economic, cultural, and environmental factors. While AD is prevalent worldwide, MENA faces distinct challenges such as rapid urbanization, aging populations, and healthcare disparities, which exacerbate the disease's impact. Both non-modifiable factors like genetics and modifiable factors, including lifestyle and environmental exposures influence AD's prevalence and severity across the region. The economic burden of AD significantly impacts family and societal structures. The need for enhanced research, early diagnosis, culturally tailored healthcare interventions, and robust public health policies to manage and mitigate AD in MENA is apparent. Applying lessons from international contexts to the MENA region and advocating for a multi-faceted approach that integrates medical, social, and policy efforts should help combat this growing health crisis effectively. This comprehensive review assesses the epidemiology, risk factors, and barriers to optimal care for AD across the MENA region, revealing a critical need for region-specific public health strategies.

## Introduction and background

While dementia encompasses a range of cognitive disorders, Alzheimer’s disease (AD) is the most prevalent and widely recognized subtype, accounting for 60-70% of all dementia cases [1]. AD currently impacts approximately 55 million people worldwide, with projections suggesting a rise to 139 million by 2050 due to aging populations and increased life expectancy [2]. Furthermore, the Global Burden of Disease study highlights dementia as the seventh leading cause of death worldwide, underscoring the need for enhanced research and healthcare strategies [3].

AD is defined by the buildup of amyloid-β plaques and neurofibrillary tangles that lead to progressive neurodegeneration [4,5]. This distinct pathology results in a relatively predictable course, beginning with memory loss and gradually advancing to severe cognitive impairment and functional decline. AD’s burden also extends beyond patients, significantly impacting caregivers, who often face emotional, financial, and social challenges. A study indicated that approximately 30-40% of dementia caregivers experience depression, anxiety, or substantial psychological distress as a result of the chronic stress associated with caregiving [6]. Financially, caregiving for AD can cost up to $34,000 annually, with nearly one in five caregivers reporting they must reduce work hours or leave employment to meet caregiving demands, often leading to long-term economic impacts on their personal finances [7]. Additionally, many caregivers encounter social sacrifices, as the extensive commitment to care responsibilities can disrupt personal relationships and prevent them from engaging in other areas of life [8]. The societal repercussions extend further as communities often face the challenge of providing adequate services and support, potentially straining local healthcare systems and affecting broader social dynamics. The Global Dementia Observatory data emphasizes that these social impacts are escalating globally, necessitating more comprehensive social support systems and community-based interventions [9].

AD’s widespread prevalence and high social and economic impact make it a critical focus for healthcare systems worldwide. While AD has been extensively studied in Western populations, there is a significant gap in research on the burden and characteristics of AD in the Middle East and North Africa (MENA) region. This review aims to bridge that gap by synthesizing data on the epidemiology, risk factors, clinical presentation, and outcomes of AD across various MENA countries. Given the distinct socio-cultural and economic context of the region, understanding AD within this framework is essential for effective public health planning. By identifying critical knowledge gaps and potential areas for intervention, this review highlights the need for targeted strategies to mitigate the growing burden of AD in MENA [10].

Review context

This literature review represents the first part of a comprehensive research initiative supported by the Fulbright U.S. Scholar Program in the United Arab Emirates. This review systematically identifies gaps and critical knowledge areas on AD within the MENA region, serving as foundational groundwork for subsequent empirical research. The second component of this initiative involves analyzing patient and hospital records in the UAE to empirically investigate the risk factors initially highlighted in this literature review, ultimately aiming to inform targeted public health strategies and interventions in the region.

## Review

Search methods

Literature Search Strategy

A comprehensive and systematic search was conducted using PubMed, Web of Science, Scopus, and Google Scholar databases. Key terms utilized included combinations of “Alzheimer’s Disease,” “dementia,” “cognitive impairment,” “Middle East,” “North Africa,” “MENA region,” “epidemiology,” “risk factors,” “prevalence,” “genetics,” “vascular factors,” “lifestyle diseases,” “environmental exposures,” “healthcare access,” “cultural influences,” “public health, ” “Algeria,” “Bahrain,” “Egypt,” “Iran,” “Iraq,” “Israel,” “Jordan,” “Kuwait,” “Lebanon,” “Libya,” “Morocco,” “Oman,” “Palestine,” “Qatar,” “Saudi Arabia,” “Syria,” “Tunisia,” "Turkey," “United Arab Emirates,” and “Yemen.” Boolean operators and advanced search functionalities were employed to optimize the accuracy and comprehensiveness of the search outcomes. Searches were limited to peer-reviewed journal articles published in English from January 1995 to December 2024, ensuring the relevance and timeliness of the reviewed literature.

Inclusion and Exclusion Criteria

Articles were eligible for inclusion if they specifically addressed AD or dementia within the MENA region or provided global insights directly relevant to the regional context. Eligible study designs included systematic reviews, meta-analyses, epidemiological surveys, observational studies, clinical trials, qualitative studies, and policy analyses. Articles were excluded if they solely discussed non-AD dementia, lacked explicit regional or cultural relevance to MENA, were inaccessible in full text, or were not peer-reviewed. Additional exclusions comprised conference abstracts, editorial commentaries, letters to editors, and non-academic sources.

Study Selection and Data Extraction

The initial search yielded approximately 140 articles. Titles and abstracts were independently screened for relevance, with subsequent full-text review conducted to verify compliance with inclusion criteria, resulting in 58 articles being selected for detailed synthesis. Data extraction emphasized thematic analysis, systematically identifying key epidemiological trends, modifiable and non-modifiable risk factors, healthcare disparities, genetic factors, cultural barriers, socioeconomic influences, and regional public health interventions pertinent to AD within the MENA context. Given the limited availability of country-specific data, global literature was selectively integrated to provide comparative context, explicitly noting these instances to highlight the regional knowledge gaps. Additional limitations include possible language biases due to the English-only inclusion criteria, potentially excluding relevant literature published in Arabic or other regional languages, as well as the inherent variability in study designs and methodologies of the included papers, which could impact the comparability and generalizability of the synthesized findings.

Epidemiology and burden of Alzheimer’s disease in MENA

**Figure 1:**
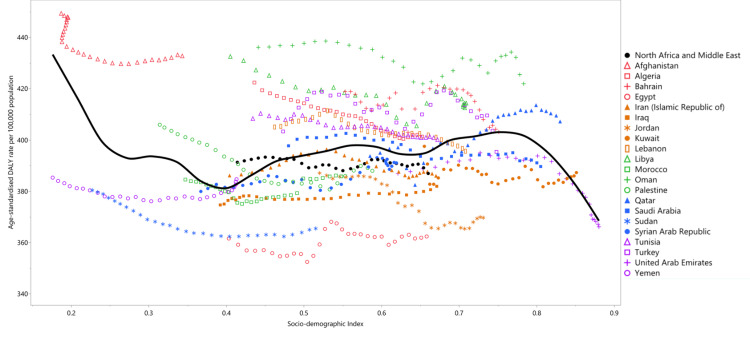
Age-standardized point prevalence rates of Alzheimer’s disease across various countries in the Middle East and North Africa region. DALY: Disability-adjusted life year. Reproduced with permission from [10].

AD and other forms of dementia have seen a steady increase in prevalence across the MENA region over the past three decades. According to Safiri et al., the age-standardized point prevalence of dementia in the MENA region was 777.6 per 100,000 people in 2019, representing a 3% increase since 1990 (Figure 1) [10]. This trend mirrors global increases in dementia prevalence, driven largely by rising life expectancy and the aging of populations [11]. Countries such as Bahrain reported some of the highest age-standardized prevalence rates, whereas the United Arab Emirates had some of the lowest [10].

Healthcare disparities across the MENA region play a critical role in AD diagnosis and treatment, pointing out that lower reported rates in some countries might be due to underdiagnosis rather than lower actual prevalence. Many low- and middle-income countries within MENA face significant barriers, including limited access to neuroimaging technologies, a shortage of trained healthcare providers, and cultural stigma surrounding dementia. In conflict-affected or lower-income areas like Yemen, for example, these challenges are amplified, leading to delayed diagnoses and limited care options [10]. By contrast, wealthier nations, such as those in the Gulf Cooperation Council (GCC), often benefit from advanced healthcare systems that facilitate earlier diagnosis and specialized care for AD. These disparities underscore the urgent need for healthcare policies that expand dementia care infrastructure, increase accessibility to diagnostic tools, and train healthcare providers to meet the demands of AD management in under-resourced areas [10,12]. In some countries, like Egypt, the usage of enhanced diagnostic practices, including hospital-based studies and widespread community screening, has contributed to a better understanding and reporting of dementia prevalence rates. This reflects not only an increase in the number of reported cases but also improvements in healthcare infrastructure and public health policies that support early detection and management of dementia [13].

Dementia cases in the region are expected to increase by 367% by 2050, far outpacing growth in high-income regions like Western Europe (74%) and the Asia Pacific (53%) [14]. Additional studies are needed to investigate the underlying causes of regional variations in AD prevalence. This would include exploring the role of genetic, environmental, and socio-economic factors, as well as healthcare access and public health policies that may contribute to these differences. Comparisons with global data and trends would provide a more comprehensive context for these regional disparities [11]. Understanding these variables will help inform future health policies and improve healthcare infrastructure where it is most needed [15].

The economic burden of dementia in MENA is substantial, with the total cost estimated to be between $10.4 billion and $13.9 billion in 2021 [16]. This figure includes both direct healthcare expenses and the costs of informal care provided by families, leading to financial strain on caregivers. Many caregivers reduce work hours or leave their jobs to care for loved ones, further adding to the economic impact of the disease and underscoring the need for targeted interventions to support affected families.

Some nations, such as Turkey and Egypt, are focusing on integrating dementia care into broader public health initiatives aimed at non-communicable diseases, while others are still developing the necessary policies and infrastructure. Adopting multisectoral approaches that incorporate healthcare, social services, and community support can reduce the burden on caregivers and improve patient outcomes across the region [17].

Risk factors for Alzheimer’s disease in MENA

AD is a complex disorder with multiple risk factors that can be categorized into non-modifiable and modifiable types. Non-modifiable factors include age, genetics, and family history. Of these, the APOE4 allele stands out as the strongest genetic risk factor, with individuals carrying two copies of the allele facing up to a 12-fold increase in the likelihood of developing AD [18,19]. APOE4 influences the accumulation of amyloid-beta in the brain, promotes neuroinflammation, and affects lipid homeostasis, all of which accelerate the onset of cognitive decline. At the same time, APOE4 is extensively studied in Western populations, but its prevalence and specific impact on MENA populations remain underexplored, warranting further genetic studies in the region [18,19]. On the other hand, modifiable risk factors, such as lifestyle choices and environmental exposures, are crucial targets for prevention and intervention strategies, particularly in a region undergoing rapid urbanization and industrialization. Up to 79% of AD risk can be attributed to genetic factors, with the remainder influenced by environmental factors and lifestyle choices [20].

Environmental Exposures

Environmental exposures, particularly air pollution, smoking, and industrialization, have also been identified as significant modifiable risk factors for AD. Exposure to high levels of air pollution has been associated with AD incidence and progression, as mostly evident from a study on regions undergoing industrialization, such as MENA [21]. Specifically, long-term exposure to fine particulate matter (PM2.5) has been linked to increased neuroinflammation, which accelerates brain aging and contributes to AD. Several studies have confirmed that individuals living in areas with high levels of pollution experience faster cognitive decline than those in cleaner environments (Table 1) [22-28]. Policies aimed at reducing air pollution and improving urban planning are essential for mitigating these environmental risks [29].

**Table 1:** Mechanisms of air pollution impact on Alzheimer's disease. PM2.5: particulate matter with a diameter of less than 2.5 micrometers; PM10: particulate matter with a diameter of less than 10 micrometers. Created independently from [22-28].

Air Pollution and Alzheimer's disease	
PM2.5 and PM10 Exposure	Exposure to PM2.5 and PM10 significantly increases the risk of developing Alzheimer's disease.
Neuropathological Changes	PM2.5 exposure is associated with severe Alzheimer's disease neuropathologic changes.
Mechanisms of Air Pollution Impact	
Neurodegeneration	Air pollution contributes to neurodegeneration, which is linked to Alzheimer's-related neurodegeneration, suggesting accelerated brain aging processes.
Blood-Brain Barrier and Immune Response	Pollutants can cross the blood-brain barrier, triggering immune responses in neurons and astrocytes, resulting in neurotoxicity and cognitive impairment.

Smoking, another modifiable risk factor, has been shown to significantly increase the risk of developing AD. A systematic review of smoking and dementia concluded that smoking contributes to both vascular and oxidative damage, further exacerbating cognitive decline [30].

Lifestyle Diseases

Lifestyle diseases such as diabetes have emerged as significant risk factors for both vascular dementia and AD. Across the MENA region, rapid urbanization and shifts in lifestyle have contributed to rising obesity and diabetes rates. Elevated blood glucose levels are associated with neuroinflammation and oxidative stress, both of which can damage neurons and accelerate cognitive decline. Even among individuals without diabetes, high glucose levels are linked to a higher risk of dementia, suggesting that glucose dysregulation plays a critical role in neurodegeneration [31]. Diabetes also contributes to amyloid plaque formation, one of the hallmark features of AD, by promoting insulin resistance in the brain. Insulin resistance impairs the brain's ability to utilize glucose efficiently, a process vital for cognitive functions such as memory and learning. This underscores the need for early intervention in diabetes management to reduce the risk of AD [32]. 

Similarly, cardiovascular diseases (CVD), including hypertension, coronary artery disease, atrial fibrillation, and heart failure, have been strongly linked to an increased risk of AD and other forms of dementia [33]. CVD contributes to cerebrovascular damage, leading to reduced blood flow to critical areas of the brain, particularly the hippocampus, which is essential for memory (Table 2) [33-35]. Over time, the chronic reduction of blood supply results in brain atrophy and cognitive decline, conditions that can progress to vascular dementia and AD [36]. Midlife hypertension accounts for up to 9.6% of dementia cases globally.

**Table 2:** Evidence for the relationship between cardiovascular and metabolic risk factors and cognitive decline or Alzheimer’s disease. AD: Alzheimer’s disease. Created independently from [33-35]. *** Strong evidence. ** Moderate evidence. * Weak evidence.

Cardiovascular Disease/Risk Factor	Evidence for Relationship with Cognitive Decline or Alzheimer’s Disease
Cardiovascular Disease	***
Hypertension	**
Lipids	**
Type 2 Diabetes	***
Apolipoprotein E Genotype	***
Inflammation	*
Moderate Alcohol Intake	***
Antioxidant Vitamins	*

Early management of these conditions significantly reduces dementia incidence, underscoring the importance of cardiovascular health in AD prevention [37]. The Lancet Commission on Dementia Prevention emphasizes that managing hypertension and other cardiovascular risk factors through lifestyle interventions, such as increasing physical activity, managing weight, and controlling blood pressure, could prevent up to 40% of dementia cases [21]. 

Additionally, comorbidity between diabetes and CVD compounds the risk, as both conditions lead to vascular and metabolic impairments that adversely affect brain health. In the MENA region, the rising prevalence of these diseases due to lifestyle changes highlights the critical need for integrated public health strategies to address these interconnected risk factors and curb the growing burden of dementia [36].

Combinatorial Effects

The interplay between modifiable risk factors and genetic predispositions in AD can intensify the effects of environmental risks on AD progression, suggesting that individuals with certain genetic backgrounds may be more vulnerable to these modifiable risks. This genetic susceptibility, combined with environmental exposures, creates a compounded risk that significantly influences the onset and progression of AD [36]. This highlights the necessity for targeted interventions that not only address environmental and lifestyle factors but also consider genetic predispositions. Understanding this relationship is vital for developing personalized approaches to prevention and treatment, which could include genetic screening and more tailored lifestyle interventions for those at higher genetic risk.

Urban Planning

An effective way to address these interconnected risks is through urban planning, which plays a crucial role in mitigating lifestyle-related risk factors for AD, particularly those linked to diabetes and CVD. Neighborhoods that promote physical activity through accessible parks, walkable paths, and social centers can encourage healthier lifestyles, which are key to managing blood sugar levels and maintaining cardiovascular health. It has been shown that environments with ample green spaces and recreational areas not only promote exercise but also reduce stress and inflammation, factors that contribute to diabetes and heart disease, both of which are significant risk factors for AD [38].

Research suggests that regular physical activity and social engagement, facilitated by well-designed urban environments, can improve insulin sensitivity and enhance cognitive resilience in older adults. A study found that urban environments designed to promote active aging were associated with lower rates of diabetes, which in turn reduced the risk of dementia [39]. The protective effects of such environments are particularly significant in regions like MENA, where urbanization has led to increased rates of sedentary behavior and metabolic disorders. Some countries within the MENA region, such as the UAE, are already implementing green urban planning initiatives and expanding public awareness campaigns, which could serve as models for dementia prevention across the region [12].

Moreover, the positive impact of urban planning extends beyond physical activity. Social infrastructure, such as community centers and cultural institutions, provides opportunities for intellectual engagement and social interaction, both of which have been shown to slow cognitive decline. Living in socially enriching environments improves cognitive health by fostering connections that reduce loneliness, a known risk factor for both CVD and dementia [40]. 

The socio-economic and lifestyle transitions observed in the MENA region are not unique; similar patterns are also being seen in rapidly urbanizing regions such as Southeast Asia and Latin America. In these regions, rising rates of diabetes, CVD, and obesity have been closely linked to urbanization, industrialization, and shifts toward more sedentary lifestyles and processed diets. For instance, countries like Mexico and Brazil have experienced a marked increase in obesity and diabetes, contributing to a growing dementia burden similar to that in MENA [15]. These findings underscore the importance of mitigating environmental and lifestyle factors to slow the progression of cognitive decline in vulnerable populations [29,37]. As these regions undergo similar transitions, global public health strategies focused on modifiable risk factors will be critical in mitigating the shared risk of AD across the developing world. This global comparison underscores the need for region-specific interventions that promote healthier lifestyles and foster social connectedness through urban design, serving as a powerful public health intervention that not only reduces the prevalence of diabetes and CVD but also lowers the risk of AD in the MENA region, while highlighting that the challenges faced by MENA are part of a broader, worldwide health issue.

Barriers to optimal care of Alzheimer’s disease in MENA 

The clinical presentation of AD in the MENA region largely aligns with global patterns, beginning with episodic memory loss and progressing to cognitive decline, along with impairments in visuospatial, speech, and executive functions [10]. These symptoms gradually lead to a loss of independence, ultimately requiring full-time care.

Healthcare Access

Access to effective treatment for AD is highly uneven across MENA, with most treatment regimens focusing on symptom management through cholinesterase inhibitors and NMDA receptor antagonists [10,13].

Such disparity in accessibility of healthcare is critical in determining AD prognosis. Wealthier nations, such as those in the GCC, benefit from advanced diagnostic tools, early interventions, and specialist care, which can slow disease progression and improve quality of life [10]. For instance, in the UAE, healthcare infrastructure supports early-stage AD diagnosis through integrated primary care programs, with approximately 75% of patients receiving diagnostic evaluations within six months of symptom onset, facilitating earlier intervention and better management [41]. These early interventions help provide comprehensive support for patients and caregivers, improving long-term outcomes [1].

In contrast, lower-income countries like Sudan and Yemen face fragmented healthcare systems that lead to delays in diagnosis, limited access to specialists, and fewer options for early intervention [12]. In Yemen, only about 20% of healthcare facilities have the capability to conduct neurocognitive assessments, reflecting broader issues with healthcare infrastructure and accessibility to specialized care [1]. Similarly, Sudanese healthcare facilities face challenges in implementing standard cognitive assessments, often due to cultural and educational barriers, leading to delays and underdiagnosis [42]. This lack of early diagnosis capabilities accelerates disease progression, placing a heavier burden on families and caregivers who may lack the necessary support and resources to manage the condition effectively. 

According to global health sources, countries with limited healthcare access face elevated DALY rates and more rapid disease progression, underscoring the critical role of healthcare accessibility in disease outcomes [1]. The disability-adjusted life years (DALYs) attributable to AD and other dementias in MENA are significantly higher than the global average, particularly in countries with lower healthcare infrastructure. For example, Iraq and Oman have DALY rates that exceed the global average by approximately 30-40% [10]. 

Cultural Factors, Education, and Community Outreach 

Beyond disparities in healthcare access, cultural and social factors significantly influence the recognition and management of AD across the MENA region. Cultural perceptions across various communities can hinder timely interventions and affect the quality of life for both patients and caregivers by reinforcing misconceptions about the condition [43,44]. Strong family support systems and the cultural norm of providing extensive household help to the elderly can inadvertently delay the recognition of early dementia symptoms. Family members often misinterpret these symptoms, leading to delayed medical consultations until more advanced stages of the disease [45]. This cultural context may mask difficulties in daily activities, a key criterion in dementia diagnosis, leading to missed opportunities for early detection and intervention. Additionally, cultural perceptions of health and illness in MENA, which often include a more spiritual and deterministic view, can contribute to stigmatization and reduce help-seeking behaviors [46]. Cultural attitudes can delay diagnosis, as memory loss is often perceived by family members as a normal part of aging rather than a clinical concern, leading to underreporting and delays in diagnosis and care [43]. In countries like Lebanon and Egypt, while resources are more accessible in urban areas, allowing for earlier diagnosis, rural populations continue to face significant diagnostic delays.

Thus, a crucial aspect of enhancing AD outcomes in the MENA region involves understanding the public's and healthcare professionals' knowledge about the disease. As previously mentioned, significant gaps in knowledge and widespread misconceptions may hinder early diagnosis and effective management of AD. For instance, a study in the Aseer region of Saudi Arabia revealed that while there is a positive attitude towards AD patients, the actual knowledge about the disease is limited, with a mean knowledge score of 10.77 out of 30, indicating a substantial gap in understanding crucial aspects of AD, including risk factors and management strategies [47]. This lack of knowledge is further complicated by the findings that demographic factors such as age and having a family history of the disease influence the level of AD-related knowledge. Younger individuals, females, and those with a family history of AD were found to have significantly better knowledge, suggesting that targeted educational interventions might be needed to address these disparities [47]. Public awareness campaigns and education on dementia can encourage early diagnosis and improve support utilization [43].

Reducing stigma also requires tailored, culturally sensitive interventions, as persistent social stigma and misconceptions continue to be significant barriers to effective care [48]. The stigma around cognitive disorders can also contribute to social isolation for patients and caregivers alike, affecting quality of life and accelerating disease progression. Campaigns in regions like South Asia have demonstrated that culturally sensitive education about mental health can significantly reduce stigma and increase healthcare engagement [16].

Cognitive assessments should also be culturally or linguistically adapted for the MENA population. Many neuropsychological tools, originally designed for Western populations, such as the Mini-Mental State Examination (MMSE), the Montreal Cognitive Assessment (MoCA), and the Addenbrooke's Cognitive Examination (ACE-III), may not adequately account for cultural, social, and linguistic differences, leading to potential misdiagnoses or underdiagnoses in these regions. There is a need for culturally sensitive neuropsychological assessments that incorporate regional linguistic and cultural nuances to ensure accurate and effective diagnosis and management of dementia [49,50].

Health organizations have recommended the development of community-based programs for early detection and intervention in AD. For example, awareness campaigns and training programs can help mitigate diagnostic delays, especially in rural and underserved regions [41]. Initiatives like those seen in parts of India, which leverage local health workers to facilitate early dementia screening, could also serve as models for MENA [51]. Morocco, for instance, started an initiative to train primary care providers in dementia diagnosis and management. Furthermore, establishing telemedicine networks could bridge access gaps by connecting remote providers with urban specialists, a model that has proven effective in other low-resource regions, such as parts of Asia and Latin America.

Gender Disparities

Gender disparities in dementia outcomes also play a notable role. Females in MENA show consistently higher rates of dementia prevalence and DALYs compared to males, influenced partly by biological factors and longer life expectancy, which raises their likelihood of developing AD as they age [10]. Data suggests that women’s dementia prevalence rates are approximately 10-15% higher than those of men globally, reflecting a combination of biological, social, and cultural factors [52,53]. Socioeconomic disparities and caregiving responsibilities also contribute to this gender gap. Previous research has discussed how caregiving burdens, often falling on women, can limit their access to healthcare and accelerate disease progression [54]. Additionally, it has been shown that sociocultural expectations and economic constraints can further exacerbate these disparities, affecting access to timely diagnosis and treatment [48].

The path forward 

Enhancing healthcare infrastructure remains a critical need to improve AD outcomes in MENA. Key steps include expanding neuroimaging access, increasing healthcare provider training in dementia care, and implementing programs to reduce the stigma surrounding cognitive disorders. Collaborative efforts, such as forming regional partnerships and working with international organizations like the World Health Organization (WHO) and Alzheimer’s Disease International, could also strengthen healthcare accessibility and infrastructure. The 1001-NAMES study by the USC Mark and Mary Stevens Neuroimaging and Informatics Institute exemplifies how research is adapting to the unique cultural and genetic backgrounds of the MENA community in the U.S., aiming to enhance the effectiveness of AD interventions. This study focuses on a range of factors, including diet, lifestyle, and genetics, specifically tailored to these communities and employs MRI scans and culturally sensitive cognitive assessments to advance understanding of AD risk factors. This holistic approach could reduce diagnostic delays and improve patient outcomes, ensuring that AD care is accessible across socio-economic boundaries [55]. The integration of comprehensive research like the 1001-NAMES study into regional health policy planning can catalyze the development of precise diagnostic and treatment methods, ultimately leading to enhanced patient care and outcomes across diverse communities.

Another key focus should be the cultural attitudes toward aging and mental health, which often delay diagnosis and treatment [43]. Addressing this requires not only public health initiatives but also culturally sensitive approaches that engage communities directly. Tailored awareness campaigns that work within the cultural framework of each country can play a crucial role in changing perceptions and encouraging early diagnosis.

The role of caregivers is another vital aspect of dementia care that requires further exploration. In MENA, caregiving responsibilities largely fall on family members, who may face emotional, physical, and financial stress. Ensuring that caregivers receive adequate support, whether through training, financial assistance, or community resources, is essential for managing the growing AD burden. Examples from Europe and North America have demonstrated the benefits of structured caregiver support programs. For example, psychoeducational interventions, which provide caregivers with training on dementia management and coping strategies, have been shown to reduce caregiver stress and improve well-being [56]. Similarly, respite care programs can offer temporary relief for caregivers, enabling them to manage their responsibilities more effectively without burnout [57]. Implementing similar programs in MENA, adapted to the region’s cultural context, could provide essential relief to caregivers and improve the overall quality of dementia care. 

The link between environmental and lifestyle factors, such as air pollution, diet, and physical inactivity, with the risk of AD, presents an opportunity for preventive strategies. The high levels of urban pollution in cities across MENA, combined with rising rates of diabetes and CVD, underscore the need for integrated public health campaigns that target these modifiable risk factors [21]. Effective urban planning that promotes physical activity and reduces pollution, alongside public education on lifestyle modifications, can play a significant role in reducing the incidence of AD. The case of Brazil, for example, sheds light on how comprehensive risk factor management and community engagement can effectively mitigate the impact of AD [58]. These strategies, once contextualized, could offer promising directions for the MENA region.

Gaps in Current Literature

A critical gap identified through this literature review is the scarcity of robust, country-specific epidemiological data on AD within the MENA region. The absence of consistent regionally adapted cognitive assessments further complicates accurate diagnosis and prevalence estimation. Limited research exploring the intersection of genetic predispositions and environmental exposures specific to this region underscores the urgent need for enhanced genetic studies tailored to diverse MENA populations. Additionally, socioeconomic disparities and rural-urban divides in healthcare access have not been sufficiently explored, highlighting a significant research void.

Recommendations for Future Research

Future research should prioritize developing culturally and linguistically adapted cognitive assessment tools tailored explicitly to the diverse MENA populations. Epidemiological studies with robust methodological designs are critically needed to generate reliable, country-specific prevalence and incidence data. Further investigation into gene-environment interactions, particularly regarding prevalent lifestyle diseases and urbanization impacts, is also essential. Addressing healthcare disparities through empirical research focusing on rural versus urban healthcare infrastructure and exploring effective, culturally sensitive community outreach programs will significantly enhance the understanding and management of AD in this region.

## Conclusions

In conclusion, managing AD in the MENA region demands a multi-faceted approach that addresses the specific cultural, socio-economic, and healthcare challenges present in each country. By improving data collection, enhancing healthcare access, supporting caregivers, and implementing targeted public health campaigns, the region can make significant strides in reducing the burden of AD. Collaboration between governments, healthcare organizations, and international bodies will be essential in developing sustainable, long-term strategies. The experiences of other regions with the integration of community-based care models and preventive health campaigns offer valuable lessons, but these must be adapted to fit the unique cultural and infrastructural landscapes of MENA.
